# Specialty preferences of studying-abroad medical students from low- and middle-income countries

**DOI:** 10.1186/s12909-023-04123-5

**Published:** 2023-03-15

**Authors:** Wen Li, Robyn M. Gillies, Chang Liu, Changhao Wu, Jiayi Chen, Xiaoning Zhang, Bin Cheng, Jing Dai, Ning Fu, Lin Li, Shenjun Liu, Hong Sun

**Affiliations:** 1 School of International Education, Xuzhou Medical University, No.209 of Tongshan Road, Yunlong District, 221004 Xuzhou, Jiangsu China; 2 School of Education, The University of Queensland, 4072 Brisbane, Australia; 3 School of Biosciences and Medicine, Faculty of Health and Medical Sciences, University of Surrey, GU2 7XH Guildford, Surrey, UK; 4 School of Management, Xuzhou Medical University, 221004 Xuzhou, China; 5 School of International Education, Weifang Medical University, 261053 Weifang, China; 6 College of International Education, Guilin Medical University, 541199 Guilin, China; 7 School of International Education, Shandong First Medical University & Shandong Academy of Medical Sciences, 271016 Tai’an, China; 8 Language and Literature School, Hebei North University, 075000 Zhangjiakou, China; 9 School of Basic Medicine, Xuzhou Medical University, Xuzhou, China

**Keywords:** High-income country, International medical student, Low- and middle-income country, Migration intention, Specialty preference

## Abstract

**Background:**

This study explored the specialty preferences of China-educated international medical students (IMSs), who are mainly from low- and middle-income countries (LMICs) and constitute a potential medical workforce both for their home countries and foreign countries, and the influence of migration intentions on their specialty preferences.

**Methods:**

A cross-sectional, questionnaire-based survey was conducted at 5 universities in China. The questionnaire link was distributed electronically among the IMSs at the 5 universities via emails. The questionnaire enquired IMSs’ demographic information, migration intentions and their specialty preferences. The Chi-square test was applied to determine the influence of the respondent’s gender, intention to practise in the home country and intention to practise in a high-income country on their specialty choices. The Chi-square test was also applied to determine the influence of the respondent’s gender, year of study and country of origin on their preferences for generalist-orientated or non-generalist orientated specialties.

**Results:**

Altogether, 452 IMSs returned their responses, yielding a response rate of 64.1%. Approximately half of the IMSs planned to not return to their home country. The most selected specialty was general surgery and the least selected specialty was physical medicine and rehabilitation. No significant differences were evident in most specialty preferences between those who intended to return home and those who intended to stay abroad. Among the IMSs having intentions of returning to their home country, male students tended to choose a generalist-orientated specialty, while female students tended to choose a non-generalist-orientated specialty.

**Conclusion:**

China-educated IMSs could play important roles in the primary care services as well as other shortage specialties both for their home countries or foreign countries. Therefore, it is recommended that governments in these countries plan migration and recruitment policies that cater for these studying-abroad medical students from LMICs, especially in this challenging time during the COVID-19 pandemic.

## Introduction

Globally, a substantial volume of students study medicine abroad, either as a priority choice or due to the failure of securing an opportunity to study in their home countries [1, 2]. Students from high-income countries (HICs) [3] and those from low-and middle-income countries (LMICs) [4] favour different education destinations though. Some Caribbean countries are among the most popular locations for the US and Canadian students studying medicine abroad [1, 5], and Central and Eastern Europe appeal more to the British, Swedish and Norwegians [6–8]. Meanwhile, some countries in Eastern Asia and Eastern Europe draw a considerable number of medical students from Africa and Southeast Asia, through medical programs that are taught either in English or in the host country’s language [2, 9, 10].

These studying-abroad students are a potential workforce for their home country’s health providers as well as the international labour market after successful completion of their overseas studies [5], although their career plans vary considerably. HIC students studying medicine abroad have been observed to commonly attempt medical licensure in their homelands to practise [11–14]. By comparison, studying-abroad LMIC students are reported to have an almost equal likelihood of returning to their home country or staying abroad in a foreign country (HIC or LMIC) to establish their medical careers [2, 10], constituting potential human resources for the world.

As the world is currently under the pressure of the COVID-19 pandemic, the issue of the shortage of medical human resources has been highlighted [15]. In fact, physician undersupply has been a long-standing global concern, whereas the demand on doctors for various specialties in different countries is in disparity regarding quantity and urgency, which are informed by demographic features, epidemiological conditions and disease burden [16, 17].

In LMICs, the scarcity of human resources for health can be critical. As per the World Health Organization (WHO), 57 of the LMICs are identified as countries with Human Resources for Health crisis [18], where doctor density is startlingly low for adequate medical services [19]. As the LMICs with human resources for health crisis are mainly rural and with a large number of indigent people, a higher density of general practitioners and family physicians is badly needed to improve healthcare access and reduce health inequality [20, 21]. However, these medically-underserved countries experience a serious deficit of generalists, attributed to factors including underdeveloped primary care-led and referral-based healthcare systems, inadequate government policy efforts for generalist physician development and retention, and doctors’ personal career preferences for specialist training [22, 23]. Despite doctors’ stronger preferences for specialist training, a lack of specialists still exists in many LMICs [16]. One key reason is that the domestic production of specialists is impeded in LMICs mainly due to the limited specialist training schemes and insufficient trainers at home [12]. This creates a trend for doctors to search for training opportunities abroad, which leads to physician emigration, exacerbating the paucity of specialists in these countries [24].

On the other side, in HICs, where the overall doctor density seems relatively satisfactory [19], human resources are also reported to be in short supply for certain medical specialties. Despite differences between countries, the persisting shortfall of physicians in general practice/family medicine (GP/FM) and psychiatry has been widely documented in many high-income economies, mainly due to medical students’ low interest in considering these specialties as a career [7, 13, 25, 26]. In addition, physician undersupply in other specialties has also been emphasised on a country-specific basis, such as pathology in Australia [27] and the UK [25], cardiology in Sweden [7], anaesthesiology in Canada [28], and vascular surgery and neurosurgery in the US [29].

Notably, the physician gaps in HICs are significantly addressed by foreign international medical graduates (IMGs) who are mainly from LMICs [30, 31]. They are known to have relatively low confidence in competition with the in-country graduates and tend to opt for less attractive specialties to enhance their chances of success in finding a position [31, 32]. In the US, foreign IMGs are largely reported to work in primary care specialities that are less popular among US medial graduates [32], providing care for unprivileged people in remote areas [33]. While the number of Canadian medical graduates who apply for family medicine programs in Canada declines, that of IMGs increases [34]. According to a study analysing all the specialty training applications in England in 2008, psychiatry was the least desirable speciality for UK graduates, whereas it had the highest proportion of applications from their international counterparts [31].

Although an extensive literature has existed on the specialty choices of medical students in LMICs, there are three aspects that are under-addressed. First, the previous studies have mainly focused on the LMIC medical students trained in their home countries, but not those trained abroad, especially in LMICs, who in fact are a considerably substantial population deserving more attention [2]. Second, given the strong emigration intentions of LMIC medical students [20, 30], their specialty choices could be affected when they prioritise finding any post in their desired country [31]. Third, there could be some bias in predicting the trend of the composition of the future domestic medical workforce across specialties based on previous studies from LMICs, if a large proportion of the participants plan to work outside their homeland.

China is accommodating over 68,000 international medical students (IMSs), mainly from LMICs [2], who may exercise their medical abilities worldwide and help balance the physician supply. Medical students often have certain preferences for and against different specialties at an early stage of their studies, even at or prior to the time of entering the medical programme [35, 36]. In this study, we investigated the specialty preferences of international LMIC students registered in medical programs in China, including their migration intentions, and explored the influence of gender, year of study and country of origin on their specialty preferences.

## Methods

### Setting and participants

We conducted a cross-sectional, questionnaire-based survey between September and October 2020, at 5 universities in China, namely Xuzhou Medical University (in Xuzhou, Jiangsu Province, east of China), Weifang Medical University (in Weifang, Shandong Province, east of China), Guilin Medical University (in Guilin, Guangxi Zhuang Autonomous Region, south of China), Shandong First Medical University & Shandong Academy of Medical Sciences (in Tai’an, Shandong Province, east of China) and Hebei North University (in Zhangjiakou, Hebei Province, north of China), which are all provincial public universities. As the majority of medical students are studying in provincial public universities in China, the type of the universities we selected is common and representative of medical studies. These 5 universities are diversified in university rankings, and are located in 5 cities across China, with different characteristics in aspects of economic levels and education resources, so that the survey results could be comparatively representative. All the available classes (a total of 705 IMSs) from the 2nd year to the final (internship) year at the participating universities were surveyed in our study. The medical programs are similar in these 5 universities, with 5-year courses of theoretical and practical studies and 1 year of rotating internship.

At the survey time, a proportion of IMSs were outside China receiving online education, as they had returned to their home countries before and during the COVID-19 pandemic and were experiencing difficulties in returning to campus due to the international travel restrictions.

#### Ethical approval

was obtained from the Ethics Committee of Xuzhou Medical University.

### Questionnaire design

The study instrument was a pre-tested, self-administered questionnaire, which comprised three sections. The first section collected students’ demographic information, such as university, gender, age, year of study, home country and current residence. The second section enquired students’ migration locations for a short-term stay (further education/training/temporary employment) and a permanent stay (stable job and life); students were asked to choose between “home country” and “a foreign country”, and indicate the name of the foreign country if they selected this option [2].

In the third section, students were required to select their first specialty choice from the specialty list. To develop the specialty list, we conducted a purposive literature review regarding medical students’ specialty choices. The selected articles were from the countries which were among the China-educated IMSs’ common countries of origin and their most desirable foreign country destinations [2]. The specialty list was formulated by reference to studies from India [37, 38], Kenya [20], Malawi [39], Nepal [36], Nigeria [40], and Pakistan [41] as well as Australia [42], Canada [43], the UK [44], and the US [45]. We also provided an “other” option for students to write down their intended specialty which was not included in the specialty list. “I do not plan to practise medicine” option was also provided. Given the possible instability in medical students’ career intentions due to the COVID-19 pandemic, an “Undecided” option was included in the current questionnaire.

As GP/FM, general internal medicine and general paediatrics are documented as generalist-oriented specialties closely related to primary care in countries among LMICs as well as HICs [46–48], we specifically clarified internal medicine and paediatrics in the specialty list. Internal medicine was divided as “(primary care) general internal medicine” to emphasise the “generalist/primary care” attribute of this specialty and “(non-primary care) internal medicine or subspecialty” to emphasise the “non-generalist/non-primary care” attribute of this specialty. The same rule was also applied to paediatrics. In addition, from the pilot study, we noticed that IMSs had different understandings of subspecialties of internal medicine by filling some of its subspecialties in “other” option (e.g. cardiology, nephrology), so we added an explanation of the main subspecialties of internal medicine [49] in the box of “internal medicine or subspecialty” in our current questionnaire. Clinical pharmacology was also added to the specialty list according to the pilot result.

### Data collection and analysis

The questionnaire link was distributed electronically among the IMSs in the 5 participating universities via emails by the authors from the respective universities, who were academics or administrators, and to facilitate a higher response rate, a reminder about the questionnaire was also sent via the online chatting tool if it was applicable. The questionnaire was in English. The aim of the study was introduced at the beginning of the questionnaire and the IMSs were also informed that their participation was voluntary. All participants gave written consent for their opinions to be published anonymously.

The obtained data were analysed using IBM SPSS Statistics (version 24.0). Specialty preferences were combined and categorised by referencing the studies of Boyd et al. [50] and Fazel & Ebmeier [31]. The Chi-square test was applied wherever applicable to determine the influence of the respondent’s gender, intention to practise in the home country and intention to practise in an HIC on each specialty preference as well as the specialty category. The Chi-square test was applied to determine the influence of the respondent’s gender, year of study and country of origin on their preferences of generalist-orientated or non-generalist orientated specialties. For the purpose of this study, students from 2nd to 3rd year were categorised into basic years and students from 4th year to final year were categorised into clinical years; students’ countries of origin were divided into Asian countries and African countries. Moreover, the Chi-square test was also applied to determine the influence of the respondents’ year of study on their specialty and migration preferences.

The level of significance (p-value) was considered significant at ≤ 0.05.

## Results

### Demographics of respondents

All together 705 respondents were invited to complete the questionnaire at the 5 universities, and 452 returned their responses. The response rate was 64.1%. Six respondents were from HICs and one respondent did not specify the home country. These responses were excluded from the data analysis. Therefore, a total of 445 responses which were completed by students from LMICs were analysed (Table 1).

**Table 1:** Questionnaire responses at the 5 universities

	A	B	C	D	E	Total	
Respondents invited (n)	288	135	112	110	60	705	
Responses returned (n)	217	89	64	42	40	452	
Responses for analysis (n)	213	88	64	42	38	445^a^	

Notes: ^a^ There were 6 students from HICs among the participating universities. Since our study focused on IMSs from LMICs, we excluded the data of these 6 students whose home countries were HICs. Another student did not clarify the home country, so this student’s data was also excluded from analysis

As shown in Table 2, 231 (51.9%) respondents were male, and 204 (45.8%) respondents were in the age group of 21–23 years. During the survey time, 333 (74.8%) respondents were staying in their home countries, 105 (23.6%) were in China and 7 (1.6%) were in other places. Most respondents (338/76.0%) were from Asian countries, and the remaining (107/24.0%) were from African countries. Among the respondents, 434 (97.5%) were from the countries facing Human Resources for Health crisis [18].

**Table 2:** Demographic data of the respondents (n = 445)

	n	%
**Gender**		
Male	231	51.9%
Female	214	48.1%
**Age**		
18–20	140	31.5%
21–23	204	45.8%
24–26	81	18.2%
≥ 27	20	4.5%
**Year of study**		
2nd year	135	30.3%
3rd year	97	21.8%
4th year	67	15.1%
5th year	65	14.6%
Final year	81	18.2%
**Current residence**		
Home country	333	74.8%
China	105	23.6%
Other place	7	1.6%
**Country of origin**		
Asian countries	338	76.0%
India	169	
Pakistan	105	
Bangladesh	37	
Nepal	23	
Afghanistan	3	
Syrian Arab Republic	1	
African countries	107	24.0%
Nigeria	24	
Ghana	13	
Zimbabwe	13	
Tanzania	11	
Zambia	10	
Gambia	5	
South Africa	5	
Somalia	4	
Malawi	3	
Burundi	2	
Ethiopia	2	
Morocco	2	
Sierra Leone	2	
Sudan	2	
Botswana	1	
Chad	1	
Comoros	1	
Congo, REP.	1	
Gabon	1	
Kenya	1	
Lesotho	1	
Mozambique	1	
South Sudan	1	

### Respondents’ specialty preferences

Table 3 describes the specialty preferences of respondents, difference by gender, intention to practise in home country and intention to practise in an HIC. The most selected specialties were general surgery (12.4%), obstetrics and gynaecology (OB/GYN) (10.8%), and GP/FM (10.3%), while the least selected specialties were physical medicine and rehabilitation (0.0%), clinical pharmacology (0.2%) and ophthalmology (0.2%). If the specialties were combined into respective groups [31, 50], surgery specialties were most chosen by the respondents (30.3%) and medicine specialties ranked the second (21.3%). Nineteen (4.3%) students did not plan to practise medicine and 13 (2.9%) did not decide their future speciality.

**Table 3:** Specialty preferences of respondents, difference by gender, intention to stay in home country and intention to stay in an HIC

Specialty preferences	All respondents				Respondents who had clear migration intentions for permanent stay					Respondents who chose a specified foreign country for permanent stay			
	Total n = 445 n (%)	Male n = 231 n (%)	Female n = 214 n (%)	p-value	Total n = 444^a^ n (%)	Those choosing home country for permanent stay n = 225 N (%)		Those choosing a foreign country for permanent stay n = 219 N (%)	p-value	Total n = 216^b^ n (%)	Those choosing an HIC n = 92 N (%)	Those choosing an LMIC n = 124 N (%)	p-value
**Surgery**	135 (30.3%)	88 (38.1%)	47 (22.0%)	0.000^*^	135 (30.4%)	64 (28.4%)	71 (32.4%)		0.363	69 (31.9%)	32 (34.8%)	37 (29.8%)	0.441
General surgery	55 (12.4%)	33 (14.3%)	22 (10.3%)	0.200	55 (12.4%)	29 (12.9%)	26 (11.9%)		0.745	26 (12.0%)	18 (19.6%)	8 (6.5%)	0.003^*^
Neurological surgery	42 (9.4%)	26 (11.3%)	16 (7.5%)	0.173	42 (9.5%)	16 (7.1%)	26 (11.9%)		0.087	24 (11.1%)	7 (7.6%)	17 (13.7%)	0.158
Cardio-thoracic surgery	14 (3.1%)	11 (4.8%)	3 (1.4%)	0.042^*^	14 (3.2%)	9 (4.0%)	5 (2.3%)		0.301	5 (2.3%)	1 (1.1%)	4 (3.2%)	0.301
Orthopaedic surgery	16 (3.6%)	13 (5.6%)	3 (1.4%)	0.017^*^	16 (3.6%)	9 (4.0%)	7 (3.0%)		0.650	7 (3.2%)	5 (5.4%)	2 (1.6%)	0.117
Plastic and reconstructive surgery	5 (1.1%)	3 (1.3%)	2 (0.9%)	0.716	5 (1.1%)	0 (0.0%)	5 (2.3%)		0.023^*^	5 (2.3%)	1 (1.1%)	4 (3.2%)	0.301
Urology	3 (0.7%)	2 (0.9%)	1 (0.5%)	0.608	3 (0.7%)	1 (0.4%)	2 (0.9%)		0.547	2 (0.9%)	0 (0.0%)	2 (1.6%)	0.221
**Medicine**	95 (21.3%)	51 (22.1%)	44 (20.6%)	0.696	94 (21.2%)	45 (20.0%)	49 (22.4%)		0.540	49 (22.7%)	18 (19.6%)	31 (25.0%)	0.346
(Primary care) General internal medicine	31 (7.0%)	21 (9.1%)	10 (4.7%)	0.067	30 (6.8%)	12 (5.3%)	18 (8.2%)		0.226	18 (8.3%)	5 (5.4%)	13 (10.5%)	0.184
(Non-primary care) Internal medicine or subspecialty	36 (8.1%)	20 (8.7%)	16 (7.5%)	0.648	36 (8.1%)	17 (7.6%)	19 (8.7%)		0.665	19 (8.8%)	10 (10.9%)	9 (7.3%)	0.354
Neurology	19 (4.3%)	9 (3.9%)	10 (4.7%)	0.686	19 (4.3%)	12 (5.3%)	7 (3.2%)		0.266	7 (3.2%)	2 (2.2%)	5 (4.0%)	0.446
Radiation oncology	8 (1.8%)	1 (0.4%)	7 (3.3%)	0.024^*^	8 (1.8%)	4 (1.8%)	4 (1.8%)		0.969	4 (1.9%)	1 (1.1%)	3 (2.4%)	0.473
Physical medicine and rehabilitation	0 (0.0%)	0 (0.0%)	0 (0.0%)	N/A^c^	0 (0.0%)	0 (0.0%)	0 (0.0%)		N/A^c^	0 (0.0%)	0 (0.0%)	0 (0.0%)	N/A^c^
Clinical pharmacology	1 (0.2%)	0 (0.0%)	1 (0.5%)	0.298	1 (0.2%)	0 (0.0%)	1 (0.5%)		0.310	1 (0.5%)	0 (0.0%)	1 (0.8%)	0.388
**Obstetrics and gynaecology or subspecialty**	48 (10.8%)	4 (1.7%)	44 (20.6%)	0.000^*^	48 (10.8%)	29 (12.9%)	19 (8.7%)		0.153	19 (8.8%)	13 (14.1%)	6 (4.8%)	0.017^*^
**General practice/Family medicine**	46 (10.3%)	32 (13.9%)	14 (6.5%)	0.011^*^	46 (10.4%)	25 (11.1%)	21 (9.6%)		0.599	20 (9.3%)	5 (5.4%)	15 (12.1%)	0.095
**Paediatrics**	32 (7.2%)	13 (5.6%)	19 (8.9%)	0.185	32 (7.2%)	16 (7.1%)		16 (7.3%)	0.937	16 (7.4%)	7 (7.6%)	9 (7.3%)	0.922
(Primary care) General paediatrics	14 (3.1%)	7 (3.0%)	7 (3.3%)	0.884	14 (3.2%)	8 (3.6%)		6 (2.7%)	0.623	6 (2.8%)	3 (3.3%)	3 (2.4%)	0.710
(Non-primary care) Paediatrics or subspecialty	18 (4.0%)	6 (2.6%)	12 (5.6%)	0.107	18 (4.1%)	8 (3.6%)		10 (4.6%)	0.589	10 (4.6%)	4 (4.3%)	6 (4.8%)	0.865
**Accident and emergency**	11 (2.5%)	5 (2.2%)	6 (2.8%)	0.664	11 (2.5%)	4 (1.8%)		7 (3.2%)	0.336	7 (3.2%)	3 (3.3%)	4 (3.2%)	0.989
Anaesthesiology	7 (1.6%)	5 (2.2%)	2 (0.9%)	0.298	7 (1.6%)	4 (1.8%)		3 (1.4%)	0.730	3 (1.4%)	1 (1.1%)	2 (1.6%)	0.744
Emergency medicine	4 (0.9%)	0 (0.0%)	4 (1.9%)	0.037^*^	4 (0.9%)	0 (0.0%)		4 (1.8%)	0.042^*^	4 (1.9%)	2 (2.2%)	2 (1.6%)	0.762
**Dermatology**	16 (3.6%)	7 (3.0%)	9 (4.2%)	0.506	16 (3.6%)	8 (3.6%)		8 (3.7%)	0.956	8 (3.7%)	4 (4.3%)	4 (3.2%)	0.666
**Psychiatry**	9 (2.0%)	4 (1.7%)	5 (2.3%)	0.651	9 (2.0%)	7 (3.1%)		2 (0.9%)	0.100	2 (0.9%)	1 (1.1%)	1 (0.8%)	0.831
**Laboratory Medicine**	6 (1.3%)	1 (0.4%)	5 (2.3%)	0.082	6 (1.4%)	2 (0.9%)		4 (1.8%)	0.392	4 (1.9%)	3 (3.3%)	1 (0.8%)	0.186
Pathology	4 (0.9%)	0 (0.0%)	4 (1.9%)	0.037^*^	4 (0.9%)	2 (0.9%)		2 (0.9%)	0.978	2 (0.9%)	2 (2.2%)	0 (0.0%)	0.099
Medical genetics	2 (0.4%)	1 (0.4%)	1 (0.5%)	0.957	2 (0.5%)	0 (0.0%)		2 (0.9%)	0.151	2 (0.9%)	1 (1.1%)	1 (0.8%)	0.831
**Community medicine and public health/Social and preventive medicine**	6 (1.3%)	1 (0.4%)	5 (2.3%)	0.082	6 (1.4%)	3 (1.3%)		3 (1.4%)	0.973	3 (1.4%)	3 (3.3%)	0 (0.0%)	0.043^*^
**Otolaryngology**	5 (1.1%)	3 (1.3%)	2 (0.9%)	0.716	5 (1.1%)	4 (1.8%)		1 (0.5%)	0.187	1 (0.5%)	1 (1.1%)	0 (0.0%)	0.245
**Radiology**	3 (0.7%)	2 (0.9%)	1 (0.5%)	0.608	3 (0.7%)	2 (0.9%)		1 (0.5%)	0.578	1 (0.5%)	0 (0.0%)	1 (0.8%)	0.388
**Ophthalmology**	1 (0.2%)	1 (0.4%)	0 (0.0%)	0.335	1 (0.2%)	1 (0.4%)		0 (0.0%)	0.323	0 (0.0%)	0 (0.0%)	0 (0.0%)	N/A^c^
**Not plan to practice medicine**	19 (4.3%)	14 (6.1%)	5 (2.3%)	0.052	19 (4.3%)	8 (3.6%)		11 (5.0%)	0.445	11 (5.1%)	2 (2.2%)	9 (7.3%)	0.093
**Undecided**	13 (2.9%)	5 (2.2%)	8 (3.7%)	0.325	13 (2.9%)	7 (3.1%)		6 (2.7%)	0.816	6 (2.8%)	0 (0.0%)	6 (4.8%)	0.032^*^

Notes: ^a^ One respondent did not have clear migration intentions for permanent stay, whose data were excluded from analysis

^b^ Three respondents who chose a foreign country for permanent stay did not specify the name of the foreign country, whose data were excluded from analysis

^c^ N/A means not applicable

Gender differences were analysed regarding respondents’ specialty preferences. Surgery ranked as the most popular specialty category for both male (38.1%) and female (22.0%) students. Medicine ranked the second for both genders (male 22.1%, female 20.6%). OB/GYN tied for the second popular specialty among female students (20.6%), while it was among the least selected specialties for male students (1.7%). After the Chi-square test, it was found that significantly more male students chose cardio-thoracic surgery (P < 0.05), orthopaedic surgery (P < 0.05) and GP/FM (P < 0.05) than female students, whereas more female students chose OB/GYN (P < 0.01), radiation oncology (P < 0.05), emergency medicine (P < 0.05) and pathology (P < 0.05) than male students.

Students’ migration intentions were found to be associated with a few of their speciality choices. Students who chose plastic and reconstructive surgery and emergency medicine were more likely to practise outside their home country (P < 0.05). Among the students who planned to practise outside their home country, students who chose general surgery (P < 0.01), OB/GYN (P < 0.05) and community medicine and public health/social and preventive medicine (P < 0.05) were more likely to practise in an HIC than an LMIC, while students who were undecided about the specialty were more likely to opted for an LMIC than an HIC (P < 0.05).

### Influence of respondents’ characteristics on their preferences of generalist-oriented or non-generalist-oriented specialties

The influence of personal characteristics on the preferences of generalist-orientated or non-generalist orientated specialties among respondents intended in returning to home country is shown in Table 4. As training in a country might lead to working in that country [20], the respondents who chose the home country either as a short-term destination or a permanent destination were all considered to have intentions of returning to their home country and thus 255 responses were included in our analysis in this comparison. After the Chi-square test, it was found that significantly more male students chose a generalist-orientated specialty while more female students chose a non-generalist-orientated specialty among this group of respondents (P < 0.01), and there were no significant differences in generalist-orientated specialty choice among the respondents of different years of study and countries of origin.

**Table 4:** Preferences of generalist-orientated or non-generalist-orientated specialties by respondents, difference by gender, year of study and country of origin

Characteristics	Students having intentions of returning to home country (n = 255^a^)			Students having intentions of going to an HIC (n = 119^b^)		
	Generalist-orientated specialties^c^	Non-generalist-orientated specialties	p-value	Generalist-orientated specialties^c^	Non-generalist-orientated specialties	p-value
**Gender**						
Male	40 (29.9%)	94 (70.1%)	0.007^*^	10 (20.0%)	40 (80.0%)	0.207
Female	19 (15.7%)	102 (84.3%)		8 (11.6%)	61 (88.4%)	
**Year of study**						
Basic years	23 (19.2%)	97 (80.8%)	0.156	9 (14.5%)	53 (85.5%)	0.846
Clinical years	36 (26.7%)	99 (73.3%)		9 (15.8%)	48 (84.2%)	
**Country of origin**						
Asian countries	44 (21.7%)	159 (78.3%)	0.274	11 (15.5%)	60 (84.5%)	0.892
African countries	15 (28.8%)	37 (71.2%)		7 (14.6%)	41 (85.4%)	

Notes: ^a^ The respondents who had intentions of returning home include the respondents who chose the home country either as the short-term destination or permanent destination. Respondents who were undecided about their specialties and those who did not plan to practise medicine were excluded from data analysis

^b^ The respondents who had intentions of going to an HIC include the respondents who chose an HIC either as the short-term destination or permanent destination. Respondents who were undecided about their specialties and those who did not plan to practise medicine were excluded from data analysis

^c^ Generalist-orientated specialties include general practice/family medicine, general internal medicine and general paediatrics

The influence of personal characteristics on the preferences of generalist-orientated or non-generalist orientated specialties among respondents intended in going to an HIC is also shown in Table 4. The respondents who chose a foreign HIC either as a short-term destination or a permanent destination were all considered to have intentions of going to an HIC and thus 119 responses were included in our analysis in this comparison. After the Chi-square test, it was found that there were no significant differences in generalist-orientated specialty choice among this group of respondents of different genders, years of studies and countries of origin.

### Influence of respondents’ year of study on specialty and migration preferences

The influence of respondents’ year of study on specialty and migration preferences is shown in Table 5. The Chi-square test showed that there were no significant differences in specialty preference among the respondents in different years of study, while there were significant differences in migration preference among the respondents in different years of study (P < 0.01). Pairwise comparison revealed that the students in fifth year were more likely to choose home country for permanent stay than the students in second year or third year.

**Table 5:** Influence of year of study on specialty and migration preferences

Preference	Second year	Third year	Fourth year	Fifth year	Final year	χ²	p-value
**Specialty preference (n = 445)**							
Generalist-orientated specialties	27 (20.0%)	15 (15.5%)	15 (22.4%)	13 (20.0%)	21 (25.9%)	17.393	0.135
Non-generalist-orientated specialties	92 (68.1%)	75 (77.3%)	51 (76.1%)	48 (73.8%)	56 (69.1%)		
Not plan to practice medicine	12 (8.9%)	2 (2.1%)	0 (0.0%)	3 (4.6%)	2 (2.5%)		
Undecided	4 (3.0%)	5 (5.2%)	1 (1.5%)	1 (1.5%)	2 (2.5%)		
**Migration preference (n = 444)** ^**a**^							
Choosing home country for permanent stay	60 (44.4%)	42 (43.8%)	31 (46.3%)	45 (69.2%)	47 (58.0%)	15.163	0.004^*^
Choosing a foreign country for permanent stay	75 (55.6%)	54 (56.3%)	36 (53.7%)	20 (30.8%)	34 (42.0%)		

Note: ^a^ One respondent did not have clear migration intentions for permanent stay, whose data were excluded from analysis

## Discussion

This study investigated the specialty preferences of IMSs at 5 universities in China. We analysed, for the first time, the influence of migration intentions of the studying-abroad medical students from LMICs on their specialty preferences. Around half (50.7%) of the students chose the home country for permanent stay and the other half (49.3%) chose a foreign country for that. No significant difference was shown in most specialty preferences between those who intended to return home and those who intended to stay abroad. This demonstrates that the studying-abroad medical students from LMICs can be an important potential medical human resource for the wider world, helping to balance the specialty mal-distribution in both LMICs and HICs.

A total of 310 IMSs indicated preference in the 4 major clinical specialties—surgery, medicine, OB/GYN, and paediatrics, which represents a majority of the respondents (69.7%). This is similar to the findings in previous studies from India [37], Nepal [22], Pakistan [41], Jordan [51], Kenya [20] and Nigeria [52], which might be related to the importance attached to these core specialties in the curriculum as well as longer durations for the internship [52]. By contrast, the proportion of IMSs who chose ophthalmology, radiology and otolaryngology was the lowest in our study, and these specialties are also selected by fewer medical students trained in other LMICs [20, 22, 37, 53–55].

GP/FM ranked the third popular specialty in our study, selected by 10.3% of IMSs as their priority choice, although it is usually reported as one of the least chosen specialties among the medical students from LMICs with very low selection percentage [22, 52, 55]. Frequently-cited reasons for the unpopularity of GP/FM include a deficiency in course provision and a lack of role models in the profession [22, 52, 53]. Therefore, the comparatively high rank of GP/FM in our study is quite surprising, as the curriculum related to GP/FM has been reportedly insufficient in medical education for IMSs in China and general practitioners/family doctors are hardly present in China’s highly-specialised hospitals where IMSs receive their clinical trainings [56]. Our finding mirrors another study from China, which found that IMSs showed a significantly more positive career attitude towards GP than Chinese medical students [57]. This suggests that the medical education environment may not be a strong factor influencing IMSs’ career intentions. Other possible factors influencing IMSs’ choice of GP/FM merit further exploration. As GP/FM is a key component for primary care services in both LMICs and HICs [26, 58, 59], IMSs’ high intent towards GP/FM regardless of their migration plans reflect the potential contribution they can make for the primary care healthcare systems globally.

Surgery was the most preferred specialty category among the IMSs in our study, supporting other reports from Asia and Africa [39, 41, 52]. However, despite the desirability of surgery as a profession, LMICs report a severe shortage of surgeons [39, 60]. This means that a significant proportion of surgical cases remain untreated [61, 62], while 6–7% of all deaths in LMICs are estimated to be avertable with basic surgical care [63]. On the other hand, it has been claimed that the shortage of surgeons not only affects resource-poor regions, but also other more affluent parts of the world [64]. Therefore, it is important to know that a large number of China-educated IMSs are ready to fill the surgical positions, mitigating the barriers to essential surgical care worldwide. Moreover, a higher preference of surgical specialties among the male students was observed in our study. The main reasons may include: first, surgery is a male-dominated field with under-representation of female surgeons [52], which is also true in the Chinese context; second, males tend to attach greater importance to prestige of the specialty while females focus more on controllable lifestyle and family responsibilities [40, 65].

Selected as the second preferred specialty choice, OB/GYN did not demonstrate any significant difference in its preference among students planning to return to their home countries and students not planning to return. However, the not-returning students tend to choose HICs over LMICs to serve as an obstetrician and gynaecologist. These qualified medical returnees would be embraced by IMSs’ home countries, where the specialists in OB/GYN are reportedly particularly scarce [66]. However, human resources in OB/GYN vary in HICs. For example, Australia, where there is an emerging shortage of doctors in OB/GYN [42], is supposed to welcome international applications in this specialty, while the UK probably rejects the international applicants as it is worrying about an oversupply of obstetricians and gynaecologists in the country [67]. We also found that female students were more likely to choose OB/GYN than male students. This has been a consistent finding in a large volume of studies from HICs as well as LMICs.

Around 2% of our respondents indicated psychiatry as their primary specialty choice, which is similar to other studies among medical students in other LMICs [53, 55, 68]. Two studies from Nigeria and Kenya respectively show an even lower preference of psychiatry, in which 0.6% and 0% of the medical students choose this specialty [20, 40]. The low number of psychiatrists in LMICs is further challenged by the brain drain, resulting in over half of psychiatrists trained in LMICs working abroad [69]. Inhabitants in lower economies are likely to suffer from mental illness related to poverty, joblessness, being less educated, deprivation and homelessness [20], but the reality is that up to 90% of people with mental disorders in LMICs have no access to basic mental healthcare [70], a shocking indication of neglect. Our study may convey a very positive signal to LMICs, as out of 9 IMSs who planned to be a psychiatrist, 7 intended to return. This suggests there is a good chance that studying-abroad LMIC medical students may return to contribute to the development of psychiatry in their home countries.

The least selected specialities, such as ophthalmology, radiology, otolaryngology anaesthesiology, attracted only a small number of our respondents, a common finding also in other studies from IMSs’ home countries, which is possibly the result of less exposure in clinical training [52, 71] and a concern about the high competition caused by the limited training opportunities. However, health workers in these specialties are badly needed in LMICs [72, 73]. It is true that the number of China-educated IMSs who prefer these specialties is not impressive, but given the paucity of doctors practising in these fields in their home countries, their return would be regarded as critically important.

We found that all the respondents who planned to practise in plastic and reconstructive surgery and emergency medicine chose to migrate abroad, which could be in connection with a concern of deficiency in further specialist training capacity in their home countries, as the resource-constrained LMICs are unlikely to provide such small-scale specialist trainings which require advanced technical equipment or operating rooms [74]. It is also worth noting that at the time of the survey, many LMICs were at the height of the COVID-19 pandemic, which may have possibly biased the results in our study, as the COVID-19 pandemic has been reported to significantly influence the specialty choice [75] as well as migration intentions [76] of medical students.

## Limitations

Firstly, this study may have been more representative of the entire group of IMSs in China if the sample size was larger with more participating universities. Secondly, although medical students do have certain specialty and migration preferences at an early stage of their studies, their attitudes could change in later years, so the responses from students in their early study years may not necessarily reflect their intent upon graduation. Finally, as participation was voluntary, there might be some bias in the results.

## Conclusion

This study has shown that China-educated IMSs have an almost equal likelihood in all their chosen specialties to practise in their home countries or abroad. This provides evidence for their great potential in supplying medical workforce and balancing doctors’ distribution in shortage specialties on a global basis. In particular, their high preference in generalist-orientated specialties demonstrates a quality desired by both HICs and LMICs. Governments in related countries are recommended to plan policies catering for these studying-abroad medical students from LMICs, who have been neglected but can play an important role in improving the healthcare outcomes, especially in this challenging time during the COVID-19 pandemic.

## Data Availability

The datasets generated and/or analysed during the current study are not publicly available due to the involved private information of the individuals, but are available from the corresponding author on reasonable request.

## Abbreviations

COVID-19: Coronavirus disease 2019
FM: family medicine
GP: general practice
HIC: high-income country
IMG: international medical graduate
IMS: international medical student
LMIC: low- and middle-income country
OB/GYN: obstetrics and gynaecology
WHO: World Health Organization.
